# Phytochemical Characterization and Biological Activities of Essential Oil from *Satureja montana* L., a Medicinal Plant Grown under the Influence of Fertilization and Planting Dates

**DOI:** 10.3390/biology13050328

**Published:** 2024-05-08

**Authors:** Hussein A. H. Said-Al Ahl, Miroslava Kačániova, Abeer A. Mahmoud, Wafaa M. Hikal, Natália Čmiková, Małgorzata Szczepanek, Karolina Błaszczyk, Siham M. Al-Balawi, Alessandro Bianchi, Slim Smaoui, Kirill G. Tkachenko

**Affiliations:** 1 Medicinal and Aromatic Plants Research Department, Pharmaceutical and Drug Industries Research Institute, National Research Centre (NRC), 33 El-Behouth St. Dokki, Giza 12622, Egypt; 2 Institute of Horticulture, Faculty of Horticulture and Landscape Engineering, Slovak University of Agriculture, Tr. A. Hlinku 2, 94976 Nitra, Slovakia; n.cmikova@gmail.com; 3 School of Medical & Health Sciences, University of Economics and Human Sciences in Warsaw, Okopowa 59, 01-043 Warszawa, Poland; 4 Department of Botany (Plant Physiology Section), Faculty of Agriculture, Cairo University, Giza 12613, Egypt; abeerkas@yahoo.com; 5 Department of Biology, Faculty of Science, University of Tabuk, Tabuk 71491, Saudi Arabia; whikal@ut.edu.sa (W.M.H.); si.albalawi@ut.edu.sa (S.M.A.-B.); 6 Department of Agronomy, Bydgoszcz University of Science and Technology, 85-796 Bydgoszcz, Poland; malgorzata.szczepanek@pbs.edu.pl (M.S.); karbla005@pbs.edu.pl (K.B.); 7 Department of Agriculture, Food and Environment, University of Pisa, Via del Borghetto 80, 56124 Pisa, Italy; alessandro.bianchi@phd.unipi.it; 8 Laboratory of Microbial Biotechnology and Engineering Enzymes (LMBEE), Center of Biotechnology of Sfax (CBS), University of Sfax-Tunisia, Sfax 3029, Tunisia; slim.smaoui@cbs.rnrt.tn; 9 Peter the Great Botanical Garden of the V.L. Komarov Botanical Institute, Russian Academy of Sciences, St. Petersburg 197376, Russia; kigatka@gmail.com

**Keywords:** *Satureja montana*, sowing date, fertilizer, essential oil, carvacrol, antimicrobial activity, antibiofilm activity

## Abstract

**Simple Summary:**

Concerted efforts by scientists and researchers are still being made to ensure the medicinal plants will be available on a continuing basis as a result of their medicinal, pharmaceutical, food, and other uses and to meet the increasing demands for them globally. *Satureja montana* is one of the multi-use medicinal plants that was introduced to Egypt to study the effect of planting dates, nitrogen, phosphorus, and fertilizers, and their interaction on the production of fresh herb and essential oil. Planting in the autumn gave the highest fresh herb, with 55 kg of nitrogen/ha and 37 kg of phosphorus/ha fertilization. For the highest essential oil production, spring planting with 55 kg of nitrogen/ha and 74 kg of phosphorus/ha was used. Much of the knowledge on its use as raw material in traditional medicine has been reported. Also, this study’s emphasis has been put on essential oil composition. This study highlights that the cultivated species belongs to *S. montana* L., a carvacrol chemotype. There is antioxidant, antimicrobial, and insecticidal activities shown by *S. montana* essential oil. Due to the limited information recorded about the influence of different agronomic factors on the plant, there needs to be agronomic studies conducted on *S. montana*, and a selection of the best ones in order to maximize its production.

**Abstract:**

The rising demand for safe plant compounds and herbal products that contribute positively to human health is in line with current market trends. Plants belonging to the *Satureja* genus, particularly the aromatic medicinal *S. montana* L. from the Lamiaceae family, are well suited to these trends as they serve as pharmaceutical raw materials. This research aimed to assess the influence of sowing date and fertilization doses, as well as their interaction, on the fresh weight, essential oil content, and composition of *S. montana*. Experimental cultivation involved varying nitrogen and phosphorus levels. The second cut had the highest fresh weight and oil production compared to the first cut. The highest total plant biomass was achieved with autumn sowing and fertilization at 55 kg N/ha and 37 kg P/ha, whereas Spring sowing exhibited higher essential oil production, with the maximum oil % with 74 kg P/ha and oil yield after applying 55 kg N/ha and 74 kg P/ha. The GC-MS analysis revealed that carvacrol was the predominant compound, with it being recommended to grow *S. montana* in Spring at doses of 55 kg N/ha and 74 kg P/ha for the superior oil yield. Additionally, *S. montana* essential oil demonstrated notable biological and antimicrobial activity, positioning it as a potential alternative to chemical food preservatives.

## 1. Introduction

Winter savory (*Satureja montana*) is a medicinal herb belonging to the Lamiaceae family, predominantly found in the Mediterranean region of Southern Europe and Northern Africa, with a presence in other global regions [1,2,3]. Highly regarded for its pharmacological activity, *S. montana* serves a multitude of purposes. It not only functions as a culinary herb, but also contributes to perfumery and serves as an active ingredient in medical preparations. Traditional medicine extensively utilizes *S. montana* for treating various conditions, including respiratory system inflammation, antiseptic needs, carminative properties, digestive aid, expectorant stimulation, gastrointestinal support, and antidiarrheic effects [4]. The prominence of essential oil in *S. montana* underscores its biological significance, driving demand for this herb in therapeutic, pharmaceutical, and industrial applications. Essential oils, especially those obtained from natural sources, have attracted growing attention from researchers due to their multifaceted benefits. The essential oil of *S. montana* stands out for its rich phytochemical composition, featuring compounds like carvacrol, thymol, β-caryophyllene, γ-terpinene, p-cymene, and linalool. These constituents provide potent antibacterial, antifungal, antiviral, antioxidant, anti-catarrhal, antitumor, antidiarrheal, stimulant, and expectorant properties, establishing. *S. montana* as a valuable resource for various health-related and industrial purposes [1,5,6,7].

The biomass and essential oil productivity of plants, as well as the composition of their essential oils, are significantly influenced by factors such as plant origin, species, environmental conditions, agronomic practices, climate, soil, harvesting period, plant part, post-harvest management, and isolation procedures [8,9]. These variables play a crucial role in shaping the plant’s biosynthetic pathways, thereby impacting the relative proportions of essential oil constituents. Achieving optimal cultivation conditions for *S. montana* is essential to ensure the production of high-quality raw material suitable for pharmaceutical purposes.

Fertilization affects plant growth and total biomass accumulation, as well as the efficiency of accumulating key bioactive compounds such as essential oils and their composition [10]. Nitrogen is widely acknowledged as the primary building block of protein and has a significant impact on plant growth. Studies have shown that nitrogen fertilizers not only increase biomass and protein accumulation, but also influence the quantity and quality of key bioactive compounds including essential oils [11,12]. Phosphorus is a crucial macronutrient involved in numerous biochemical processes that impact plant yield components [13]. In addition, it plays a key role in the metabolism of secondary compounds in plants [10,14]. While the effects of macronutrients like nitrogen and phosphorus on key herbaceous traits are well understood, less attention has been given to the effects of fertilization with each element individually or in combination, as well as the dosages, on *S. montana* production.

Studies have demonstrated the significant impact of planting dates on plant growth, biomass production, and essential oil yield. Weather conditions, particularly temperature, play a crucial role in plant growth and yield by influencing metabolic activities such as photosynthesis, respiration, and transpiration. Variations in herb and essential oil yields have been linked to changes in photosynthetic activities [15]. Consequently, harvest timing has received considerable attention as an important agronomic trait due to its effect on herb and oil yields, as well as essential oil composition [11,16].

*S. montana* is recognized as a promising industrial crop, particularly as it emerges as a newly cultivated medicinal plant in Egypt. Therefore, there is a scarcity of research on *S. montana* in Egypt. The optimal agrotechnical factors for *S. montana* and their influence on the productivity of fresh herb and essential oil have not been extensively studied. With no available data on planting dates and its important role in plant productivity, as well as its effect on essential oil of the *S. montana*, they have not been studied before. Moreover, there is no information concerning its nutrient requirements in Egypt; only one study conducted by Said-Al Ahl and Hussien [4] reported the positive effect of nitrogen and phosphorus fertilization on the yield of dry herb and essential oil. The aim of this study is to determine appropriate production strategies for the *S. montana* carvacrol chemotype to effectively manage supply and demand in the global markets for carvacrol-bearing essential oils. However, further studies are needed to fully address this objective.

This study aimed to evaluate the effects of various nitrogen and phosphorus doses, both individually and in combination, along with different planting dates, on the fresh herb biomass, essential oil yield, and its composition in *S. montana*. An innovative aspect of this research was the exploration of the combined impact of nitrogen and phosphorus fertilization with optimal planting dates (autumn and spring) to enhance *S. montana* productivity under Egyptian conditions, which represents a novel area of investigation for *S. montana* plants. Additionally, the study investigated the in vitro and in situ antimicrobial, antibiofilm, and insecticidal activities of the essential oil.

## 2. Materials and Methods

### 2.1. Site Description 

This research was conducted at the experimental farm of the Faculty of Pharmacy, Cairo University, located in Giza governorate, Egypt. Prior to sowing, the soil’s physical and chemical characteristics were assessed following the method outlined by Jackson [17]. The soil was classified as sandy loam, with the following physical composition: 51.1% sand, 25% silt, 23.9% clay, and 0.47% organic matter. The soil’s chemical properties were determined as follows: Electrical Conductivity (E.C) = 4.3 ds/m, pH = 7.85, and total nitrogen (N), available phosphorus (P), and exchangeable potassium (K) content were 0.07, 0.53, and 2.8 mg/kg, respectively [17].

### 2.2. Plant Materials and Experimental Design

*Satureja montana* seeds, sourced from HEM ZADEN B.V (Venhuizen, Netherlands) underwent nursery sowing on 15 October and 15 March for winter and summer planting, respectively, across two consecutive growing seasons. Following a two-month period in the nursery, uniform seedlings were transplanted into the field on 15 December (autumn sowing) and 15 May (spring sowing) in both seasons into 3 × 3.5 m plots on rows with 60 cm apart and 20 cm between seedlings; the seedlings were thinned 30 days after being transplanted to leave one plant per hill to give 85 plants/plot. The seedlings were irrigated, and the soil was kept moist. The study employed a factorial design, implemented in a complete randomized block with three replications. Phosphorus treatments ((P0) = 0, (P1) = 37, and (P2) = 74 kg P/ha, as calcium superphosphate (P_2_O_5_, 15.5%)), were applied prior to transplanting on both sowing dates. Nitrogen application, as a dressing application ((N0) = 0, (N1) = 55, and (N2) = 110 kg N/ha, as urea (46% N), was divided into two equal portions at 30 and 45 days from transplanting. The fertilizer treatments are T1(N0P0), T2(N55P0), T3(N110P0), T4(N0P37), T5(N0P74), T6(N55P37), T7(N55P74), T8(N110P37), and T9(N110P74). All other recommended agricultural practices were uniformly applied during the period of the experiments.

### 2.3. Measurement of Fresh Weight and Oil Yield

On July 15 and September 15 of both seasons, plant harvesting occurred, and the fresh herb was weighed (g/plant). Essential oil extraction from the fresh herb was determined by hydro-distillation for 3 h of each experimental unit according to Guenther [18]. Essential oil content (%) was quantified as ml per 100 g of fresh herb, and essential oil yield was expressed as ml per plant by multiplying the oil content (%) by the average yield per plant of fresh herb. For each experimental unit, three internal replications were hydro-distilled and their average was used for statistical analysis. The collected essential oils underwent dehydration over anhydrous sodium sulfate and were stored in a refrigerator until subjected to GC-MS analyses.

### 2.4. GC-MS Analyses and Identification of Components

The GC-MS analysis was performed using a TRACE GC Ultra Gas Chromatograph (Thermo Fisher Scientific, Waltham, MA, USA) coupled with a THERMO mass spectrometer detector (ISQ Single Quadrupole Mass Spectrometer, Thermo Fisher Scientific, Waltham, MA, USA). A TGWAX MS column (30 m × 0.25 mm i.d., 0.25 μm film thickness) was employed in the GC/MS system. Helium was used as the carrier gas at a flow rate of 1.0 mL/min with a split ratio of 1:10. The temperature program was composed of an initial 1 min at 40 °C, followed by a ramping at 4.0 °C/min to 160 °C (held for 6 min), and then a further increase at 6.0 °C/min to 210 °C (held for 1 min). Both the injector and detector temperatures were maintained at 210 °C. Diluted samples (1:10 hexane, *v*/*v*) of 0.2 μL were consistently injected. Mass spectra, obtained via electron ionization (EI) at 70 eV in the *m*/*z* range of 40–450, were used for compound identification, utilizing authentic chemicals, the Wiley spectral library collection, and the NIST library [19,20].

### 2.5. Antioxidant Potential 

This method was adapted from a previous study [21]. The antioxidant activity of essential oil was assessed using the method of radical scavenging with 2,2-diphenyl-1-picrylhydrazyl (DPPH, Sigma Aldrich, Germany). A solution of DPPH (0.025 g/L dissolved in methanol) was adjusted to an absorbance of 0.7 at a wavelength of 515 nm. In a 96-well microplate, 5 μL of essential oil sample was added to 195 μL of DPPH solution, and the reaction mixture was incubated for 30 min in the dark with continuous shaking at 1000 rpm. The antioxidant activity was quantified as the percentage of DPPH inhibition using the formula (A0 − AA)/A0 × 100, where A0 represents the absorbance of DPPH and AA represents the absorbance of the sample. To standardize the results, antioxidant activity was compared to a standard reference, Trolox (Sigma Aldrich, Schnelldorf, Germany), dissolved in methanol (Uvasol^®^ for spectroscopy, Merck, Darmstadt, Germany) within a concentration range of 0–100 µg/mL.

### 2.6. Antimicrobial Assay

#### 2.6.1. Tested Microorganisms

The essential oil’s antimicrobial effectiveness was assessed against various strains of bacteria and yeast. Gram-positive bacteria included *Micrococcus luteus* CCM 732, *Listeria monocytogenes* CCM 4699, and *Staphylococcus aureus* subsp. *aureus* CCM 3953, while Gram-negative bacteria included *Escherichia coli* CCM 3953, *Salmonella enterica* subsp. *enterica* CCM 3807, and *Yersinia enterocolitica* CCM 7204T. Yeast strains included *Candida albicans* CCM 8186, *C. glabrata* CCM 8270, *C. krusei* CCM 8271, and *C. tropicalis* CCM 8223. All microbial strains were obtained from the Czech Collection of Microorganisms in Brno, Czech Republic. Prior to analysis, bacterial and yeast cultures were incubated for 24 h at 37 °C and 25 °C in Mueller–Hinton Broth (MHB, Oxoid, Basingstoke, UK) and Sabouraud Dextrose Broth (SDB, Oxoid, Basingstoke, UK), respectively. The bacterial and yeast inoculum’s optical density was standardized to 0.5 McFarland standard on the day of the experiment [22].

#### 2.6.2. Disk Diffusion Method

The disc diffusion sensitivity test used the same method as in a previous study [22]. The antimicrobial activity of the microorganisms listed in Section 2.6.1 was evaluated. Mueller–Hinton Agar (MHA) and Sabouraud Dextrose Agar (SDA) were utilized for culturing bacterial and yeast strains, respectively. Discs impregnated with *S. montana* essential oil were placed on the agar surface. After 24 h of incubation at 37 °C for bacteria and 25 °C for yeast, the inhibition zones were measured. Positive controls (gentamicin, cefoxitin, and fluconazole) were included, and each experiment was conducted in triplicate [22].

#### 2.6.3. Minimal Inhibition Concentration

The determination of minimal inhibitory concentration (MIC_50_ and MIC_90_) values followed the methodology by Kačániová et al. [23]. A 96-well microtiter plate was filled with 50 μL of microbial inoculum, and *S. montana* essential oil was added at various concentrations in Mueller–Hinton Broth (MHB). Negative and positive control wells were established, and plates were incubated for 24 h at 37 °C for bacteria and 25 °C for yeast. Absorbance at 570 nm was measured using a spectrophotometer (Glomax, Promega Inc., Madison, WI, USA). MIC_50_ and MIC_90_ values, representing the concentrations inhibiting 50% and 90% of bacterial growth, respectively, were determined. The procedure was repeated three times for reliability [23].

#### 2.6.4. In Situ Analyses on the Vegetable

In this study, we investigated the antibacterial properties of *S. montana* essential oil in the vapor phase and its ability to inhibit the growth of yeasts and bacteria. To ensure proper sealing, we applied MHA and SDA to both the lid and the bottom of Petri dishes. The kohlrabi (fresh bought from a regular supermarket) was cut into 0.5 mm rounds and served as a food model, rinsed three times in distilled water. Using disposable bacterial eyelets, selected bacteria were introduced into the kohlrabi, and yeasts were cultured on MHA and SDA. To assess *S. montana* essential oil’s effects, filter paper was cut to fit the diameter (60 mm) of the Petri dish lid, and 100 μL of different *S. montana* essential oil concentrations (diluted in ethyl acetate to achieve 62.5, 125, 250, and 500 μg/mL) were pipetted onto the paper [24]. The control group received ethyl acetate treatment. The Petri dishes containing yeast and bacteria were then incubated at 25 °C and 37 °C for seven days in a dark, hermetically sealed environment. Three replicates were performed for each measurement. Using stereological analysis, we determined the inhibition of microorganism growth. We employed ImageJ software 1.50i (The National Institutes of Health, Bethesda, MD, USA) to calculate the volumetric density (Vv) of the microbes. The stereological network points for the colony (P) and substrate (p) were counted. The microorganism’s growth density was calculated as a percentage using the formula Vv = P/p × 100. The growth inhibition of microorganisms (MGI) as a percentage was used to express the *S. montana* essential oil’s antimicrobial activity in vapor form, calculated as MGI = [(C − T)/C] × 100, where C represents the growth density of microorganisms in the control group and T represents the growth density of microorganisms in the treated group.

### 2.7. Biofilm Development Study

#### Crystal Violet Assay

The assessment of Minimal Biofilm Inhibitory Concentration (MBIC) followed the methodology outlined by Kačániová et al. [23]. Bacterial suspensions were incubated in Mueller–Hinton Broth (MHB, Oxoid, Basingstoke, UK) for 24 h at 37 °C in an aerobic environment. An inoculum with an optical density of 0.5 McFarland standard was prepared, and a microtiter plate with 96 wells was utilized. Each well received 50 μL of the prepared inoculum and 100 μL of MHB, with the first column of the microplate receiving 100 μL of the essential oil. A two-fold dilution resulted in concentrations from 100 mg/mL to 0.049 mg/mL. Maximal growth control used MHB with a bacterial inoculum, while MHB with essential oil served as the negative control. After a 24 h incubation period at 37 °C, the absorbance was measured at 570 nm using the Glomax spectrophotometer (Promega Inc., Madison, WI, USA). The MBIC, defined as the concentration at which the absorbance equaled or was less than the negative control, was determined. The concentrations inhibiting 50% and 90% of biofilm development were designated as MBIC_50_ and MBIC_90_. This process was repeated for validation [23].

### 2.8. Insecticidal Activity

*Harmonia axyridis* was employed as the model organism for assessing *S. montana* essential oil insecticidal activity. Groups of 100 individuals were exposed to various *S. montana* essential oil concentrations (100, 50, 25, 12.5, 6.25, and 3.125%) on filter paper in Petri dishes. After 24 h, living and deceased individuals were counted. The experiment was conducted in triplicate to validate results [22].

### 2.9. Statistical Analysis

The acquired data underwent analysis involving mean and standard deviation. Normal distribution was confirmed through the Shapiro–Wilk test. Multi-variate analysis of variance (MANOVA) was employed to scrutinize fresh weight (g), oil content per 100 g of fresh weight (mL/100 g FW), and oil yield per plant (mL/plant). The study explored the impact of N and P fertilization, sowing date, and their interactions. Tuckey’s HSD test assessed mean differences at a significance level of *p* = 0.05. Statistical calculations utilized the Statistical 13.0 PL package (Stat soft, Poland). Graphs illustrating the fresh weight, oil content (mL/100 g FW), and oil yield (mL/plant) of *S. montana*, encompassing the first and second cuts, were generated using Grapher 21 (Golden Software, Golden, CO, USA). For antimicrobial activity assessments, conducted in triplicate, mean values ± standard deviation (SD) was reported. One-way ANOVA, followed by the Tukey’s HSD test at *p* < 0.05 significance, was performed using CoStat version 6.3 (Cohort software, Santa USA).

## 3. Results

### 3.1. Fresh Weight 

Our study confirmed the impact of sowing date (*p* < 0.001), fertilization doses (*p* < 0.001), and their interaction (*p* < 0.001) on the fresh weight of *S. montana* plants during the first and second cuts (Supplementary Materials, Table S1; Table 1). Specifically, *S. montana* sown in autumn exhibited a significant increase in herb fresh weight compared to those sown in spring in both cuts. *S. montana* fresh weight was increased with increasing fertilization doses of nitrogen and/or phosphorus. In general, plants fertilized with 55 kg N/ha and 37 kg P/ha gave higher herb fresh weight compared to other doses. The maximum plant weight in the first cut was achieved with fertilization doses of 55 kg N/ha and 37 kg P/ha (Table 1). In the second cut, 55 kg N/ha and 37 kg P/ha and 55 kg N/ha and 74 kg P/ha gave a higher fresh weight.

**Figure 1:**
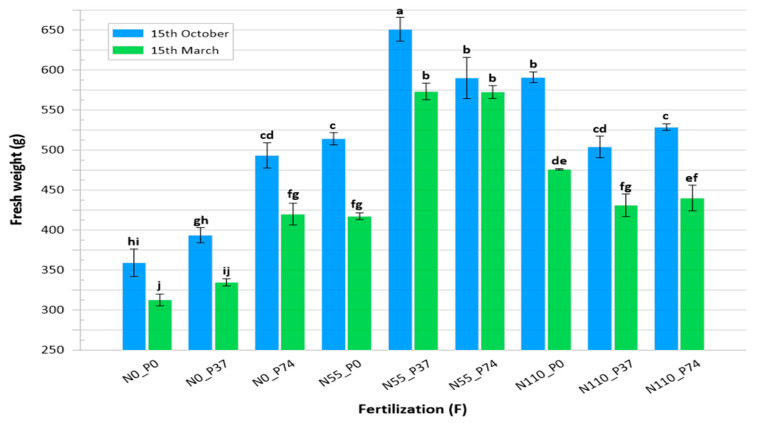
Fresh weight of *Satureja montana* (g per plant) as affected by sowing date and NP fertilization, sum from the first and second cut, on average from the first and the second growing seasons. Data are the mean (±SD) of 3 samples. ^a–j^ Different letters refer to significant differences for D × F (Tukey, *p* < 0.05).

Regarding the interaction treatments between planting dates and fertilization doses, the interaction treatments showed a significant clear effect on herb fresh weight compared to unfertilized plants. Plants sowing in autumn and fertilized with 55 kg N/ha and 37 kg P/ha gave the maximum values (245.4 and 405.6 g per plant) during the first and second cuts, respectively. As a result of our findings, we recommend that planting *S. montana* plants in autumn and fertilizing with 55 kg N/ha and 37 kg P/ha to obtain the highest fresh herb productivity (Figure 1).

### 3.2. Essential Oil Content 

The results about essential oil content indicate a significant influence of sowing date (*p* < 0.001), fertilization doses (*p* < 0.001), and their interaction (*p* < 0.001) on the essential oil content (%) of *S. montana* plants (Supplementary Materials, Table S1; Table 2).

Our investigations demonstrated that the oil content (mL/100 g FW) in *S. montana* plants was significantly higher in spring sowing than in autumn sowing in the first and second cuts (Table 2). *S. montana* essential oil was increased with increasing fertilization doses of nitrogen and/or phosphorus. Phosphorus fertilization showed the most significant impact on increasing the oil content compared to nitrogen fertilization alone or in combination with phosphorus. As the phosphorus fertilization doses increased, so did the essential oil content. Interestingly, it was observed that fertilizing with nitrogen at 55 kg/ha resulted in a higher oil percentage compared to the higher dose of 110 kg/ha. Moreover, the oil content in plants was higher during the second cut compared to the first cut. It is worth noting that the oil content in plants, during both the first and second cuts for both autumn and spring sowings, was lowest in the treatment without fertilization.

Regarding the interaction treatments between planting dates and fertilization doses, the interaction treatments showed a significant clear effect on essential oil compared to unfertilized plants. Plants sowing in spring and fertilized with 74 kg P/ha only gave the maximum values (0.438 and 0.458 mL/100 g FW) during the first and second cuts, respectively. The maximum essential oil content was achieved by sowing in spring with phosphorus fertilization at 74 kg/ha during the second cut.

### 3.3. Essential Oil Yield

The effect of sowing date, fertilization doses, and their interaction on the oil yield of *S. montana* was also found to be significant (Supplementary Material, Table S1). Essential oil yield of *S. montana* was higher in the second cut compared to the first cut (Table 3). A significant increase in oil yield was found when *S. montana* was planted in the autumn compared to when it was planted in the spring during the first cut. On the other hand, there is a conflict during the second cut, i.e., there was a significant increase in oil yield in plants planted through spring compared to autumn planting.

There was a difference in the oil yield with respect to the fertilization treatments. It was found that, plants fertilized with 55 kg N/ha and 74 kg P/ha, gave the highest oil yield (0.729 and 1.426 mL per plant FW) in the first and second cuts, respectively. By increasing the fertilization doses of nitrogen and/or phosphorus, the oil yield increased. Control lants that were not fertilized gave the lowest values in both cuts.

**Figure 2:**
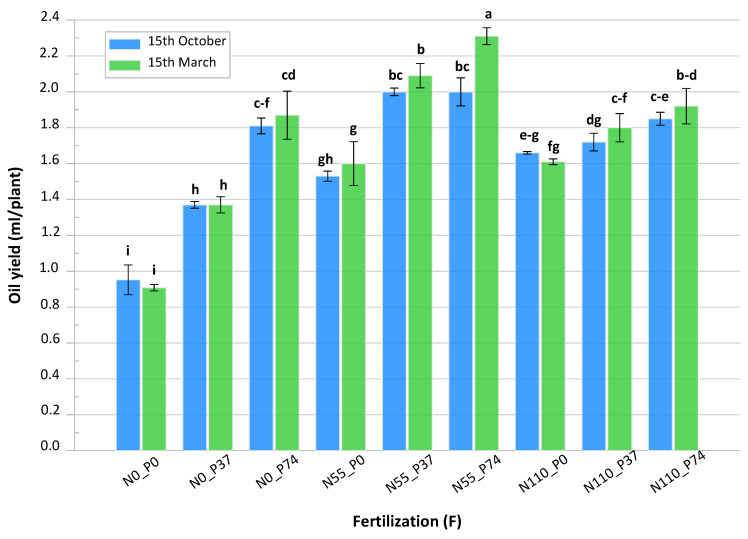
Oil yield (ml/per plant FW) of *Satureja montana* as affected by sowing date and NP fertilization, sum from the first and second cut, on average from the first and the second growing seasons. Data are the mean (±SD) of three samples. ^a–i^ Different letters refer to significant differences for D × F (Tukey, *p* < 0.05).

Regarding the interaction treatments between planting dates and fertilization doses, the interaction treatments showed a significant difference in essential oil yield for most treatments. Plants sowing in spring and fertilized with 55 kg N/ha and 74 kg P/ha gave the maximum values (0.762 and 1.553 mL per plant FW) during the first and second cuts, respectively. It is also clear from Figure 2 that the highest essential oil yield (1.553 mL per plant FW) was obtained from sowing *S. montana* in the spring and fertilization with 55 kg n/ha and 74 kg p/ha in the second cut.

### 3.4. Essential Oil Composition 

The detailed composition of essential oil isolated from *S. montana* in our study is provided in tables (Table 4, Tables S2 and S3). The analysis reveals that the essential oil extracted from fresh *S. montana* herb predominantly contains carvacrol as its major compound, constituting over 10% of the oil. Additionally, there are five minor compounds, including thymol, p-cymene, γ-terpinene, β-caryophyllene, and linalool, each with concentrations exceeding 1% but less than 10% (Table 4). Compounds with concentrations less than 1% are traces, as shown in Table S2. It is also clearly shown that four main chemical groups can be distinguished based on their chemical properties: monoterpene hydrocarbons, oxygenated monoterpenes, sesquiterpene hydrocarbons, and oxygenated sesquiterpenes. Oxygenated monoterpenes and monoterpene hydrocarbons constitute the largest share, ranging from 69.84% to 84.71% and 8.97% to 19.98% for both autumn and spring sowings (Table S3). 

Carvacrol was found to be the most abundant compound in *S. montana* during both autumn and spring sowings, followed by thymol, another significant oxygenated monoterpene. Linalool ranked third among the terpenes identified (Table 4). Monoterpene hydrocarbons, including p-cymene and γ-terpinene, were the most abundant in plants sown both in October and March (Table 4). Additionally, our study identified β-caryophyllene, a sesquiterpene hydrocarbon, as an important compound (Table 4). 

However, the applications of N, P, and their interaction fertilizers appeared to exert a more pronounced influence on the essential oil composition, especially in the case of high concentration compounds like carvacrol, p-cymene, γ-terpinene, and thymol (Table 4). Although phosphate fertilization had greater effect than nitrogen, while the interaction treatments between nitrogen and phosphorus had the highest effect, and accordingly the application of the highest dosages of N and P (110 and 74 kg/ha, respectively) resulted in the greatest quantity of carvacrol (77.35%, 76.67%) and thymol (5.87%, 4.76%) during autumn and spring planting dates, respectively. The control plants contained the lowest amount of carvacrol (65.32%, 65.41%) and thymol (2.56%, 1.97%) during autumn and spring planting dates, respectively. Conversely, linalool and the most significant monoterpene hydrocarbons, p-cymene, and γ-terpinene have the opposite behavior, with its highest concentration observed in control plants (without fertilization) and the lowest concentration was obtained from the highest dosages of N and P (110 and 74 kg/ha) compared to the control plants (Table 4). For β-caryophyllene, there was no specific effect for fertilizer on its content.

According to Table 4 and Table S3, the total percentage of oxygenated compounds exceeded that of non-oxygenated compounds, and this ratio varied depending on the applied parameters. The highest proportion of oxygenated compounds (94.13%) was observed in the essential oils of plants fertilized with N110 + P74 kg/ha, while the lowest proportion (86.67%) was found in the essential oils of non-fertilized plants (control). This trend was consistent with the distribution of carvacrol. It was noted that between 86.67% and 94.13% of the identified compounds were monoterpenes, with the remaining compounds being sesquiterpenes, the proportions of which varied according to the treatments applied.

### 3.5. Antioxidant Activity

The assessment of the antioxidant potential of *S. montana* essential oil relied on its ability to counteract the stable cation DPPH radical. The findings revealed a significant inhibition of 69.37% by *S. montana* essential oil against the DPPH radical.

### 3.6. Antimicrobial Activity

This study investigated the inhibitory effects of *S. montana* essential oil on the growth of different bacterial and fungal strains, employing disc diffusion and minimal inhibitory concentration (MIC) methods. Interestingly, among Gram-positive bacteria, *S. aureus* displayed the highest level of resistance, while *L. monocytogenes* showed the greatest susceptibility. Among the Gram-negative bacterial strains, *E. coli* demonstrated the greatest resistance, while *S. enterica* and *Y. enterocolitica* showed heightened sensitivity. Additionally, the study unveiled significant antimicrobial activity of *S. montana* essential oil against the yeast strain *C. krusei*, as outlined in Table 5.

After obtaining the results from the disk diffusion method, further investigation of the antibacterial properties of *S. montana* essential oil was carried out using microdiffusion experiments. The results, including the minimum inhibitory concentrations (MIC_50_ and MIC_90_), are shown in Table 6. Among Gram-positive bacterial strains, *S. aureus* showed the lowest MIC_50_ (2.19 mg/mL) and MIC_90_ (2.35 mg/mL) values, while *M. luteus* exhibited the highest MIC_50_ and MIC_90_ values. In Gram-negative bacteria, *Y. enterocolitica* was the most sensitive strain, with an MIC_50_ of 18.43 mg/mL and an MIC_90_ of 18.65 mg/mL, while *E. coli* displayed the highest resistance, with an MIC_50_ of 13.16 mg/mL and an MIC_90_ of 13.37 mg/mL. Yeasts demonstrated varying MIC against *C. albicans* (MIC_50_ of 22.18 mg/mL and MIC_90_ of 22.35 mg/mL) and *C. glabrata* (MIC_50_ of 23.43 mg/mL and MIC_90_ of 23.74 mg/mL). Gram-positive bacteria exhibited higher sensitivity to *S. montana* essential oil, with an MIC_50_ range of 2.19 to 3.34 mg/mL, compared to Gram-negative bacteria, which showed a higher range of 13.16 to 18.43 mg/mL. The study indicated that very low concentrations of the essential oil effectively inhibited all tested strains, showcasing strong antibacterial activity.

*S. montana* essential oil exhibited significant antibiofilm activity against *S. enterica*, with a MBIC_50_ of 4.56 mg/mL and a MBIC90 of 4.79 μg/mL, as determined through the crystal violet technique (Table 6). The effectiveness of *S. montana* essential oil in preventing biofilm formation or eliminating preexisting biofilm by strains of *Staphylococcus aureus*, *Escherichia coli*, and *Listeria monocytogenes* was assessed using the crystal violet staining method. Specifically, concentrations of 1/4 and 1/8 MIC of *S. montana* essential oil were employed to evaluate the impact of *S. montana* essential oil on bacterial biofilm formation activity. Increased concentrations of the essential oil led to a notable decrease in biofilm formation across all strains examined. Particularly, the *L. monocytogenes* strain displayed a dose-dependent decrease, suggesting that higher concentrations of *S. montana* essential oil were linked to a corresponding reduction in biofilm formation.

Our study aimed to explore the effectiveness of *S. montana* essential oil in the vapor phase. Gram-positive, Gram-negative, and yeast bacteria were cultivated on kohlrabi to assess the impact of *S. montana* essential oil, as outlined in Table 7. In a kohlrabi model contaminated with Gram-positive bacteria, the most effective concentration of *S. montana* essential oil against *S. aureus* (86.46%) was observed at a concentration of 62.5 µg/mL. Additionally, in the vapor phase against the Gram-negative bacterial strain, 62.5 µg/mL was identified as the most effective concentration in inhibiting the growth of *Y. enterocolitica* (96.37%). For *C. albicans* and *C. krusei* in the kohlrabi model, *S. montana* essential oil exhibited maximum efficacy at a concentration of 62.5 µg/mL, achieving inhibition rates of 76.56% and 76.43%, respectively. 

The insecticidal activity of *S. montana* essential oil against *H. axyridis* was evaluated, and the results are presented in Table 8. The data indicate that the highest insecticidal activity of the assessed essential oil was observed at concentrations of 50% and 100%. However, at concentrations of 6.25% and 3.15%, *S. montana* essential oil did not exhibit significant repellent properties against *H. axyridis*.

## 4. Discussion

Fertilization plays a crucial role in providing the necessary elements for plant growth and development. Ninou et al. [25] reported that nitrogen fertilizer increased plant growth and biomass production of Greek oregano. Further studies on *S. hortensis* have corroborated the beneficial effects of nitrogen fertilization, leading to a notable enhancement in plant growth and overall herbage yield [26,27]. This stimulation, as reported by Abd-Allah et al. [28], is achieved through the activation of photosynthetic pigments and enzymatic reactions within the plant. Nitrogen plays a crucial role in plant biology, as it contributes to the synthesis of essential compounds such as nucleic acids, proteins, phytohormones, and chlorophyll. These substances are vital for various cellular processes, including growth and biomass production. Additionally, nitrogen facilitates proton and electron transport during photosynthesis and respiration, which are fundamental metabolic processes in plants. Moreover, it is involved in signaling pathways that enable communication between different organs within the plant [29,30]. Also, an adequate nitrogen source is essential for increasing in phosphorus uptake may be attributed to rhizosphere acidification [31] or the mobilization of soil phosphorus, enhancing phosphorus uptake efficiency by the roots [32]. Lima et al. [33], in their study on *Ocimum kilimandscharicum* plants, reported that plant growth and yield were positively influenced by phosphorus addition. Phosphorus is essential for plant growth and development, serving as a critical component of the plant nucleus and protoplast. It plays a vital role in the formation of nucleic acids and phosphoesters, which are essential for various cellular processes. Additionally, phosphorus is involved in the production of adenosine triphosphate (ATP), which serves as an energy currency for the plant. Furthermore, phosphorus supports root development, promotes flower bud differentiation, and facilitates flowering, contributing to overall plant health and reproductive success [29]. Additionally, morphological characteristics and mass production exhibited significant increases with higher P doses [34,35,36,37]. On *Satureja khuzestanica*, Nooshkam et al. [38] observed that the dry weight increased with the application of (50 N and 30 P kg/ha) or (100 and 60 kg/ha). Previous studies reported that nitrogen and phosphorus fertilization, alone or in combination, has a positive effect on morphological parameters and herb productivity [34,35,36].

Overall, there is a lack of research addressing the impact of planting dates, as well as nitrogen and phosphorus fertilization, on the productivity of *S. montana* or other *Satureja* species. Specifically, there have been no prior studies investigating the influence of autumn and spring planting dates on *S. montana*. This highlights the novelty and importance of the current research in expanding our understanding of the optimal cultivation practices for *S. montana*. A study conducted on *S. sahendica* revealed that the choice of sowing dates, whether in autumn or spring, had a significant impact on various plant attributes, including the number of tillers, plant height, the quantity of lateral branches on the main stem, and the yield of individual plant shoots [39]. Notably, autumn planting out-performed spring sowing in all the mentioned traits. Similarly, research conducted by Righini et al. [40] on *Camelina sativa* emphasized the advantages of autumn sowing dates, correlating them with increased plant aboveground biomass.

The limited available information highlights the need for further exploration into the fertilization practices for optimal cultivation of *Satureja* plants. Sahzabi et al. [41] conducted a study on summer savory, showing that the application of soil nitrogen effectively promotes plant growth, resulting in higher shoot yield. Additionally, the application of 100 kg/ha phosphorus was found to increase the fresh weight yield compared to control plants [42]. The application of phosphorus fertilizer, as observed by Said-Al Ahl and Abdou [10] significantly influenced the growth parameters of dragonhead. Alharbi et al. [13] demonstrated that phosphorus application led to increased fresh biomass in parsley cultivars. Additionally, research by Mehanna et al. [43] confirmed that simultaneous application of phosphorus fertilizer significantly enhanced various plant growth characteristics, including plant height, number of branches, number of umbels per plant, and seed yield in coriander. Additionally, a single study by Said-Al Ahl and Hussien [4] indicated that the individual or combined application of nitrogen and phosphorus fertilizers significantly increased the herb dry weight of *S. montana*. Our study reinforces these findings, highlighting the importance of nitrogen and phosphorus fertilizer application rates in influencing the biomass production of *S. montana*.

Previous research indicated that the essential oil % in *S. montana* dried herb varied from 0.22 to 2.80% depending on the origin [4,30,44,45,46,47]. Sowing date has a significant impact on the percentage of essential oil in *Satureja* species. A study on *S. sahendica* revealed that the oil concentration was markedly higher in autumn sowing compared to spring sowing [39]. Piccaglia et al. [48] noted that peppermint plants planted in autumn displayed higher essential oil percentages and oil yields compared to those planted in spring. Similarly, in the case of *S. montana*, plants fertilized with NP in the second cut exhibited the highest oil yield compared to the first cut, which was attributed to increased herb weight and essential oil content. [4]. The formation of essential oils in medicinal and aromatic plants typically fluctuates based on crucial factors, contributing to significant variations in essential oil as temperature, day-light hours, light intensity, promoting increased photosynthesis as well as soil conditions, and the growth period [46,47,49,50,51]. Specifically, spring cultivation (February and March) resulted in the highest essential percentage in the first cut, while autumn cultivation (October and November) during both seasons yielded the highest percentage for *R. officinalis* in the second cut under the study conditions [52].

Prior investigations have revealed the effect of fertilization on oil content. Nooshkam et al. [38] observed a higher oil concentration in *S. khuzestanica* plants with 50 kg N/ha and 30 kg P/ha compared to the dose 50 kg N/ha and 30 kg P/ha. Abd-Allah et al. [28] found a positive effect of high NPK doses (476, 238, and 119 kg/ha) on oil concentration in *S. hortensis*. Essential oil content displayed an increasing trend with elevated nitrogen or phosphorus application rates, consistent with previous findings, indicating that increased N or P fertilization can enhance essential oil production in certain aromatic plants [25,26,27,34,35,36]. Phosphorus might influence enzymes within the mevalonate pathway, crucial for terpene biosynthesis [53], and, consequently, essential oil production appears to be impacted by P fertilization [54]. Nitrogen fertilization plays a significant role in essential oil biosynthesis by influencing plant metabolism and contributing to the synthesis of plant constituents through various enzymes. Baranauskienne et al. [55] observed that nitrogen fertilizer increased the essential content of thyme and basil. These findings align with previous research indicating that the addition of phosphate and nitrogen fertilization, or their interaction, has a positive effect on essential oil content [43,55,56]. 

Prior research highlights that achieving a high oil yield in the cultivation of *Satureja* species is closely linked to either high biomass production or elevated essential concentration [39,44,57,58]. The increased biomass production and essential oil percentage in autumn sowing compared to spring positively influenced the oil yield of *S. sahendica* [39]. Studies involving *S. khuzestanica* demonstrated that fertilization dose (50 N and 30 P kg/ha) led to a higher essential content and yield compared to higher doses (100 N and 60 P kg/ha) [38]. Additionally, in other investigations on this species, NPK fertilization (50-25-25 kg/ha) also spurred the essential oil percentage and yield of *S. macrantha* [59]. Compared to the control, there was a positive trend in essential oil yield with increasing rates of N and P application, indicating that the application of N and P fertilizers enhanced essential oil production in *S. montana*. This finding is consistent with previous research, suggesting that increased N and P fertilization contributes to higher essential oil production in certain aromatic plant [4,25,26,27,34,35,36], where the greatest increase in essential oil formation is due to phosphorus fertilizer. A plausible explanation for this phenomenon lies in the fact that plant terpenoids are synthesized through a biosynthetic pathway utilizing acetyl coenzyme A as a crucial substrate [59,60], and since P is a constituent element of acetyl-coenzyme A, elevating the P fertilizer amount can stimulate the biosynthesis of plant terpenoids, consequently boosting essential oil yield in *S. montana* plants.

Our findings align with previous research that identified carvacrol, p-cymene, γ-terpinene, and β-caryophyllene as the predominant compounds in *S. montana* [1,53]. Kulić et al. [61] reported that the essential of *S. montana* from Herzegovina was composed of carvacrol (54.9%), γ-terpinene (14.5%), p-cymene (8.8%), β-caryophyllene (3.2%), linalool (1.2%), and thymol (0.3%). However, variations in the composition of essential oils are influenced by factors such as the altitude of plant growth, the time of collection during the season, and the variability of *S. montana* genotypes [45,62]. 

A review of the literature indicates substantial variations in the relative concentrations of principal components, including carvacrol, thymol, linalool, γ-terpinene, and p-cymene in *S. montana* essential oil due to the existence of different chemotypes [63]. Our study revealed that carvacrol was the predominant compound of the essential oil of *S. montana* cultivated in Egypt, as previously confirmed by [4,64], up to 80%, and so this indicates the promising antioxidant and antimicrobial properties of *S. montana* essential oil, and from here, *S. montana* essential oil of Egyptian origin belongs to the carvacrol chemotype and is considered an important agent for potential biological activities.

Piccaglia et al. [48] observed no impact of planting date on oil composition in peppermint plants. In our study, as shown in Table 4, we noticed an increase in carvacrol content when planting in spring compared to autumn. However, the sowing date did not have a clear impact on the composition of linalool, γ-terpinene, β-caryophellene, p-cymene, and thymol compounds.

Previous studies on *S. montana* have highlighted that ecological factors, characteristic of growth habitats, significantly impact the quantitative and qualitative chemical composition of the essential oil [1,5,6]. Piccaglia and Marotti [48] noted that variations in the relative amounts of thymol, carvacrol, γ-terpinene, and p-cymene in the *S. montana* essential oil grown in Italy were attributed to the effects of environmental conditions. Kustrak et al. [65] observed variations in oil composition content from plants collected at ten ecologically distinct locations in Croatia, Bosnia, and Herzegovina. The major constituents were carvacrol (30.83–84.19%), thymol (0.45–13.61%), p-cymene (3.36–26.28%), γ-terpinene (0.16–15.87%), linalool (0–11.49%), and β-caryophyllene (0–10.68%). Mirjana and Nada [47] found that carvacrol was the main constituent, especially before flowering, while p-cymene increased through flowering in *S. montana*. It has also been shown that γ-terpinene and p-cymene are the biogenetic precursors of thymol and carvacrol, and that there is a negative relationship between γ-terpinene and carvacrol [11].

The impact of nitrogen and phosphorus fertilization on the essential oil composition of *S. montana* has not been extensively studied. One notable study by Said-Al Ahl and Hussien [4] delved into this, focusing on the chemical composition of essential oils and how it is affected by NP fertilizer. Their findings highlighted changes in the levels of carvacrol, thymol, and their precursors p-cymene and γ-terpinene due to NP fertilizer. Specifically, they observed a decrease in the contents of p-cymene and γ-terpinene compounds with fertilization, while carvacrol and thymol showed an increase. However, the behavior of β-caryophyllene with fertilization was not as clearcut. On the other hand, the second cut gave the highest content of carvacrol, thymol, and β-caryophyllene, while the highest content of p-cymene and γ-terpinene was obtained at the first cut. The highest percentage of carvacrol (80.39%) recorded with fertilization dose (50 Kg N + 30 Kg P/fed.) was in the second cut.

Research on *S. hortensis* has demonstrated that increased nitrogen fertilization can have an effect on essential oil content and its compositions; the highest carvacrol content was noted for nitrogen dose (4 g N/m) [12]. Nitrogen fertilizer increased the essential oil content and differences in its compositions percentages in *Dracocephalum moldavica* and *Plectranthus amboinicus* [56,66]. The authors emphasized that nitrogen directly influences terpene synthesis in plants by being a component of structures involved in the biosynthesis process. Additionally, nitrogen has indirect effects through its impact on photosynthesis, which in turn influences terpene production. This is especially significant in the promotion of carvacrol synthesis, a compound influenced by nitrogen, which results in a reduction in monoterpene hydrocarbons [11,67], a finding that is consistent with our study results. 

Additionally, Lima et al. [33] observed differences in oil compounds in *Ocimum kilimandscharicum* due to phosphorus application. Peng and Ng [34] observed that increasing P fertilization rates led to higher concentrations of β-caryophyllene in the essential oil of *Vitex negundo*. Also, phosphorus fertilization causes an increase in the essential oil content and differences in the percentages of the compounds of these essential oils for *Dracocephalum moldavica*, *Petroselinum crispum*, and *Coriandrum sativum* [10,13,43].

Bakhtiari et al. [57] observed alterations in the essential oil composition of *S. macrantha* upon the application of NPK fertilizers, particularly influencing the levels of thymol. Mohammadi et al. [58] suggested that biofertilizers and organic fertilizers (NPK) enhanced the percentage and phenolic compounds (carvacrol) in the essential oil of *S. khuzistanica*. Peng and Ng [34] also reached the same result that fertilization (NPK) increases essential oils and bioactive compounds in *Vitex negundo*. As mentioned previously, nitrogen and phosphorus play crucial roles in various biochemical pathways responsible for the synthesis of important plant compounds, including secondary metabolites. The synergistic action of N and P may contribute to more pronounced effects on these processes, potentially accounting for substantial changes in essential oil composition [67]. Nitrogen and phosphorus are essential structural components of proteins, phospholipids, and coenzymes structures, and also participate in various physiological metabolisms of plants and biosynthesis of secondary metabolites. P is used in the biosynthesis of secondary metabolites such as monoterpenes, sesquiterpenes, and diterpenes, components of essential oils [34]. 

The chemical composition of *S. montana* essential oil appears to be influenced by factors such as geographic origin and environmental conditions, particularly temperature and light exposure. Additionally, fertilization practices have a noticeable impact on the makeup of essential oils. These findings suggest that the quantity, method, and type of fertilization should be carefully adjusted to optimize the biosynthesis of secondary metabolites and consequently enhance the content of the desired bioactive components. 

*S. montana* under study in Egypt is classified as a carvacrol chemotype as it is the only main compound whose percentage exceeds 80% [4,64]. The importance of carvacrol as an oxygenated monoterpenes that has known antimicrobial, anti-septic, and antioxidant agents with low acute toxicity and weak genotoxic potential [4,68]. The Food and Drug Administration (FDA) and the European Commission (EC) have approved carvacrol as an additive [68]. We recommended cultivation *S. montana* L., a carvacrol chemotype in Egypt, as a new and important source of carvacrol used as potent agents in food preservation and for the therapeutic or nutraceutical industries.

In a study by Marin et al. [67], *S. montana* was collected from various regions, and its essential oil was extracted, revealing significant antioxidant activity measured at 4.21 mg/mL, surpassing results obtained from *S. montana* samples collected in Bosnia and Herzegovina. The essential oils of many *Satureja* species, including thymol and carvacrol, exhibit antimicrobial activity, making *S. montana* a promising source for the production of food products with antioxidant effects and herbal preparations with antimicrobial and antioxidant activities [69,70]. In a study by Tumbas et al. [71], various extracts derived from *S. montana* essential oil, including n-butanol, methanol, and water extracts, showed significant antioxidant effects against DPPH radicals at concentrations of 0.20 and 0.30 mg/mL. These extracts demonstrated antioxidative activity equivalent to 100%. On the other hand, the ethyl acetate extract did not display notable antioxidative effects at concentrations ranging from 0.05 to 0.15 mg/mL. However, at higher concentrations (0.15 to 0.3 mg/mL), the ethyl acetate extract showed improved antioxidative activity, with antioxidative activity values ranging from 16.88% to 32.5%. Mihajilov-Krstev et al. [44] also reported that *S. montana* essential oil displayed antimicrobial, antioxidant activity, and antifungal compounds. In a study by Lin et al. [72], they tested 42 of the most commonly used essential oils. Among them, cinnamon bark (91.4 ± 0.002%), origanum (86.66 ± 0.008%), and wild thyme (52.54 ± 0.016%) showed the strongest DPPH free-radical scavenging activity at a concentration of 5 mg/mL. In our work, *S. montana* essential oil showed 69.37% activity against DPPH radical, which is among the second most potent EO among all the EOs tested compared to the reported study.

Our results are consistent with prior research highlighting the antibacterial efficacy of *S. montana* essential oil against various microorganisms, attributed to the potent antimicrobial properties of carvacrol and thymol [73,74,75]. Pino-Otín et al. [76], in their study on *S. montana*, documented the bactericidal properties against a broad spectrum of Gram-positive and Gram-negative pathogenic bacteria, along with fungicidal effects on *C. albicans*. The essential oil, particularly rich in carvacrol and thymol, demonstrated significant efficacy against priority 1 bacteria, such as *Pseudomonas aerogenes*, *Streptococcus agalactiae*, and *Acinetobacter baumannii*, as designated by the World Health Organization (WHO). Given the antimicrobial efficacy of *S. montana* essential oil and ethanol extracts, they could serve as valuable ingredients in medicinal formulations such as oral liquids, foodborne disease treatments, and sprays for wound care, oral infections, and other infectious ailments [5]. Further research has highlighted the abundance of essential oils in *S. montana*, particularly carvacrol and thymol, which are oxygen-containing phenolic monoterpenes [77]. These compounds, notably carvacrol and thymol isomers, found in winter savory, exhibit a range of beneficial biological activities [78,79]. Vladic et al. [80] concluded that carvacrol content ranged from 71% to 82%, with an average of 78.61%. Furthermore, carvacrol has been confirmed as an antitumor agent. The antibacterial efficacy of essential oils has been extensively examined using direct contact antimicrobial assays, including diffusion and dilution techniques, confirming their well-established antibacterial properties against various pathogens [81]. The study conducted by Mihajilov-Krstev et al. [44] found that Gram-positive bacteria, including *S. aureus*, exhibited greater susceptibility to *S. montana* essential oil. Ćavar et al. [82] observed varying levels of resistance in different bacterial strains, with *P. aeruginosa* displaying higher resistance. Carramiñana et al. [83] demonstrated the efficacy of *S. montana* essential oil against clinical entero-pathogens such as *Vibrio parahaemolyticus*, *S. enterica* serovar *Typhimurium*, *Escherichia coli*, *Plesiomonas shigelloides*, *Shigella flexneri*, and *Yersinia enterocolitica*. Our findings support the sensitivity of *S. aureus* to *S. montana* essential oil, aligning with previous studies on Gram-positive bacteria. Our research further validates the established efficacy of *S. montana* essential oil in inhibiting the growth and survival of *Listeria monocytogenes* [83] and preventing food contamination from *Bacillus cereus* [84]. Additionally, the observed antibacterial properties against pathogenic and spoilage yeasts, as highlighted by Ciani et al. [85], underscore the broad-spectrum antimicrobial activity of *S. montana* essential oil. Notably, *S. montana* essential oil demonstrates potent antimicrobial activity against both Gram-positive (*Listeria monocytogenes*, *Staphylococcus aureus*, and *Staphylococcus haemolyticus*) and Gram-negative clinical isolates (*Escherichia coli*, *Klebsiella pneumoniae*, *Pseudomonas aeruginosa*, and *Serratia marcescens*) [1].

Our findings, in line with Vitanza et al. [84], highlight the potential of *S. montana* essential oil to effectively inhibit biofilm formation and disrupt preexisting biofilm, particularly demonstrating a dose-dependent effect against *L. monocytogenes*. Pino-Otín et al. [76] suggest that *S. montana* oil could serve as an alternative to conventional antibiotics.

With growing concerns about the safety of food additives, consumers are increasingly turning to natural options to replace chemical preservatives. These alternatives include plant extracts, in particular essential oils, which are gaining attention for their potential use in food preservation. Essential oils derived from plants, which are generally considered safe, are becoming increasingly popular due to their widespread acceptance among consumers and their consistency with the growing demand for natural alternatives [86,87]. These compounds are considered safe for application in food products, and their use as preservatives is driven by their antibacterial, antifungal, and antioxidant properties, providing a natural alternative to synthetic preservatives [86,87,88,89].

Numerous species within the *Satureja* genus, such as *S. aintabensis* Davis, *S. bachtiarica* Bung, *S. cilicica* Davis, *S. cuneifolia* Ten, *S. hellenica* Halásky, *S. hortensis* L., and *S. intermedia*, have shown promising potential. Evaluations of *S. montana* essential oil have demonstrated their efficacy against a diverse range of arthropods, including mites, ticks, and plant-harming nematodes. These insects belong to various orders, such as Coleoptera, Diptera, Hemiptera, Homoptera, Lepidoptera, Phthiraptera, and Thysanoptera [90].

## 5. Conclusions

In our investigation of *S. montana*, we established the impact of sowing date and NP fertilization doses on fresh weight, essential oil, and its composition. In fact, a significant effect of planting date on the fresh weight was observed during the two cuts. Fresh weight values were significantly higher when *S. montana* planted in autumn compared to planted in spring. Also, there was significant effect from planting date and fertilizer treatments observed in the first cut, but not significant during the second cut. The opposite result was obtained regarding to oil content (%), while in the oil yield, there was a discrepancy between the first and second cuts, as planting in the autumn gave a significant increase in the first cut, while planting in March gave a significant increase in the second cut. Higher total plant biomass was obtained by sowing in autumn with N fertilization at 55 kg/ha and P fertilization at 37 kg/ha (245.4 and 405.6 fresh weight, g per plant) during the first and second cuts, respectively. However, under phosphorus fertilization, increasing P application rates can effectively promote essential oil production; furthermore, the application of 74 kg P kg/ha has the optimal essential oil content (0.458 and 0.458%) in March sowing during the first and second cuts. The highest total oil yield (1.553 mL per plant) resulted from spring sowing with 55 kg N/ha and 74 kg P/ha at the second cut. Carvacrol was the predominant compound of the essential oil of *S. montana* cultivated in Egypt, up to 80%. Its percentage increased with increasing NP fertilization, reaching the highest (76.67 and 77.37%) of the highest dose of fertilization (110 kg N/ha and 74 kg P/ha) during autumn and spring planting, respectively. *S. montana* essential oil is biologically effective, as a result of carvacrol, with thymol, p-cymene, and γ-terpinene compounds show promise as antioxidants, antimicrobial agents, and anti-insect agents. As a final consideration emerging from the study, *S. montana* essential oil exhibited notable antioxidant activity. The most potent in vitro antimicrobial effect was observed against Gram-positive *S. aureus*, and the in situ method on the kohlrabi model demonstrated efficacy against all three Gram-negative bacteria. *S. montana* essential oil emerges as a potential method for extending the shelf-life of vegetables. Therefore, we recommend the cultivation of *S. montana* L., especially the chemotype with carvacrol, as a valuable medicinal plant that has recently begun to be cultivated in Egypt. For optimum results, we recommend autumn planting with nitrogen fertilization at 55 kg/ha and phosphorus fertilization at 37 kg/ha in order to obtain the highest yield of fresh herb. Conversely, spring sowing combined with nitrogen fertilization at 55 kg/ha and phosphorus fertilization at 74 kg/ha is recommended to achieve the highest yield of essential oil. Further agricultural research on *S. montana* is warranted to maximize fresh herb and essential oil production under Egyptian conditions. In addition, investigation of its diverse chemical constituents may increase its biological importance and expand its use in the pharmaceutical, nutritional, and cosmetic fields.

## Figures and Tables

**Table 1:** Fresh weight (FW) (g per plant) of *S. montana* as affected by sowing date and NP fertilization in the first and second cut, on average from the first and the second growing seasons.

Fertilization (F)	Cut					
	1st Cut			2nd Cut		
	Date of Sowing (D)					
	October	March	Mean	October	March	Mean
N0_P0	140.5 ± 8.73 ^f–h^	102.5 ± 8.12 ^i^	121.5 ± 22.2 ^F^	218.6 ± 9.19 ^f^	210.2 ± 0.884 ^f^	214.4 ± 7.43 ^F^
N0_P37	145.8 ± 4.92 ^fg^	112.7 ± 1.70 ^ij^	129.2 ± 18.5 ^F^	247.9 ± 5.23 ^e^	222.0 ± 3.01 ^ef^	235.0 ± 14.7 ^E^
N0_P74	185.9 ± 5.28 ^e^	118.2 ± 9.81 ^ij^	151.5 ± 38.3 ^E^	307.6 ± 10.6 ^cd^	302.7 ± 4.40 ^cd^	305.1 ± 7.72 ^CD^
N55_P0	205.4 ± 7.95 ^c–e^	128.7 ± 5.84 ^g–i^	167.0 ± 42.5 ^D^	308.8 ± 6.29 ^cd^	288.7 ± 4.03 ^d^	298.7 ± 12.0 ^D^
N55_P37	245.4 ± 7.33 ^a^	199.5 ± 9.41 ^c–e^	222.4 ± 26.3 ^A^	405.6 ± 14.5 ^a^	373.9 ± 1.58 ^b^	389.7 ± 19.6 ^A^
N55_P74	214.2 ± 10.0 ^bc^	192.1 ± 2.07 ^de^	203.2 ± 13.7 ^B^	375.9 ± 22.2 ^b^	380.5 ± 8.00 ^ab^	378.2 ± 15.2 ^A^
N110_P0	228.3 ± 3.52 ^ab^	150.8 ± 4.31 ^f^	189.5 ± 42.6 ^C^	362.7 ± 7.00 ^b^	325.2 ± 4.18 ^c^	343.9 ± 21.1 ^B^
N110_P37	196.5 ± 3.69 ^c–e^	120.9 ± 4.43 ^h–j^	158.7 ± 41.5 ^DE^	307.6 ± 10.4 ^cd^	310.1 ± 9.78 ^cd^	308.8 ± 9.12 ^CD^
N110_P74	206.6 ± 1.53 ^cd^	127.2 ± 8.08 ^g–i^	166.9 ± 43.8 ^D^	322.1 ± 2.83 ^c^	313.0 ± 8.18 ^cd^	317.5 ± 7.41 ^C^
Mean	196.5 ± 33.9 ^A^	139.0 ± 33.9 ^B^	167.8 ± 44.4	317.4 ± 57.7 ^A^	302.9 ± 56.1 ^B^	310.2 ± 56.8

Data are the mean (±SD) of three samples. ^A–F^ Different uppercase letters refer to significant differences for D or F (Tukey, *p* < 0.05); ^a–j^ Different lowercase letters refer to significant differences for D × F (Tukey, *p* < 0.05).

**Table 2:** Oil content in fresh herb (mL/100 g FW) of *S. montana* as affected by sowing date and NP fertilization in the first and second cut, on average from the first and the second growing seasons.

Fertilization (F)	Cut					
	1st Cut			2nd Cut		
	Date of Sowing (D)					
	October	March	Mean	October	March	Mean
N0_P0	0.238 ± 0.018 ^h^	0.267 ± 0.008 ^g^	0.253 ± 0.020 ^E^	0.282 ± 0.008 ^i^	0.302 ± 0.013 ^g–i^	0.292 ± 0.014 ^G^
N0_P37	0.288 ± 0.008 ^fg^	0.365 ± 0.009 ^c^	0.327 ± 0.043 ^C^	0.383 ± 0.014 ^cd^	0.433 ± 0.012 ^ab^	0.408 ± 0.030 ^AB^
N0_P74	0.333 ± 0.003 ^d^	0.438 ± 0.003 ^a^	0.386 ± 0.058 ^A^	0.387 ± 0.006 ^cd^	0.458 ± 0.008 ^a^	0.423 ± 0.040 ^A^
N55_P0	0.283 ± 0.006 ^fg^	0.370 ± 0.009 ^c^	0.327 ± 0.048 ^C^	0.307 ± 0.012 ^f–i^	0.387 ± 0.046 ^cd^	0.347 ± 0.053 ^EF^
N55_P37	0.272 ± 0.008 ^fg^	0.332 ± 0.003 ^d^	0.302 ± 0.033 ^D^	0.330 ± 0.009 ^e–h^	0.383 ± 0.008 ^cd^	0.357 ± 0.030 ^DE^
N55_P74	0.325 ± 0.009 ^d^	0.397 ± 0.006 ^b^	0.361 ± 0.040 ^B^	0.345 ± 0.005 ^d–g^	0.408 ± 0.015 ^bc^	0.377 ± 0.036 ^CD^
N110_P0	0.265 ± 0.005 ^g^	0.315 ± 0.010 ^de^	0.290 ± 0.028 ^D^	0.290 ± 0.000 ^hi^	0.350 ± 0.005 ^d–f^	0.320 ± 0.033 ^F^
N110_P37	0.318 ± 0.003 ^de^	0.405 ± 0.005 ^b^	0.362 ± 0.048 ^B^	0.357 ± 0.006 ^de^	0.423 ± 0.010 ^a–c^	0.390 ± 0.037 ^BC^
N110_P74	0.297 ± 0.015 ^ef^	0.410 ± 0.010 ^b^	0.353 ± 0.063 ^B^	0.383 ± 0.003 ^cd^	0.447 ± 0.010 ^ab^	0.415 ± 0.035 ^AB^
Mean	0.291 ± 0.031 ^B^	0.367 ± 0.052 ^A^	0.329 ± 0.057	0.340 ± 0.040 ^B^	0.399 ± 0.050 ^A^	0.370 ± 0.054

Data are the mean (±SD) of three samples. ^A–G^ Different uppercase letters refer to significant differences for D or F (Tukey, *p* < 0.05); ^a–i^ Different lowercase letters refer to significant differences for D × F (Tukey, *p* < 0.05).

**Table 3:** Oil yield (mL per plant FW) of *S. montana* as affected by sowing date and NP fertilization in the first and second cut, on average from the first and the second growing seasons.

Fertilization (F)	Cut					
	1st Cut			2nd Cut		
	Date of Sowing (D)					
	October	March	Mean	October	March	Mean
N0_P0	0.336 ± 0.044 ^hi^	0.274 ± 0.023 ^i^	0.305 ± 0.046 ^E^	0.616 ± 0.039 ^h^	0.634 ± 0.027 ^h^	0.625 ± 0.031 ^G^
N0_P37	0.420 ± 0.004 ^f–h^	0.411 ± 0.015 ^gh^	0.415 ± 0.011 ^D^	0.949 ± 0.017 ^g^	0.960 ± 0.034 ^g^	0.954 ± 0.025 ^F^
N0_P74	0.619 ± 0.016 ^b–d^	0.484 ± 0.097 ^e–g^	0.551 ± 0.097 ^C^	1.187 ± 0.029 ^d–f^	1.387 ± 0.037 ^bc^	1.287 ± 0.114 ^CD^
N55_P0	0.582 ± 0.011 ^c–e^	0.476 ± 0.027 ^fg^	0.529 ± 0.061 ^C^	0.947 ± 0.017 ^g^	1.120 ± 0.144 ^ef^	1.034 ± 0.132 ^EF^
N55_P37	0.665 ± 0.005 ^a–c^	0.661 ± 0.037 ^a–c^	0.663 ± 0.024 ^B^	1.339 ± 0.018 ^b–d^	1.434 ± 0.031 ^ab^	1.386 ± 0.056 ^AB^
N55_P74	0.696 ± 0.037 ^ab^	0.762 ± 0.004 ^a^	0.729 ± 0.043 ^A^	1.299 ± 0.089 ^b–d^	1.553 ± 0.044 ^a^	1.426 ± 0.152 ^A^
N110_P0	0.605 ± 0.015 ^b–d^	0.474 ± 0.015 ^fg^	0.540 ± 0.073 ^C^	1.052 ± 0.020 ^fg^	1.138 ± 0.029 ^ef^	1.095 ± 0.052 ^E^
N110_P37	0.625 ± 0.017 ^b–d^	0.490 ± 0.024 ^e–g^	0.558 ± 0.077 ^C^	1.097 ± 0.037 ^e–g^	1.313 ± 0.057 ^b–d^	1.205 ± 0.126 ^D^
N110_P74	0.613 ± 0.035 ^b–d^	0.522 ± 0.045 ^d–f^	0.567 ± 0.061 ^C^	1.235 ± 0.018 ^c–e^	1.399 ± 0.060 ^ab^	1.317 ± 0.098 ^BC^
Mean	0.573 ± 0.115 ^A^	0.506 ± 0.138 ^B^	0.540 ± 0.130	1.080 ± 0.218 ^B^	1.215 ± 0.279 ^A^	1.148 ± 0.257

Data are the mean (±SD) of three samples. ^A–G^ Different uppercase letters refer to significant differences for D or F (Tukey, *p* < 0.05); ^a–i^ Different lowercase letters refer to significant differences for D × F (Tukey, *p* < 0.05).

**Table 4:** Effect of sowing date and fertilization with N and P, as well as their combination, on the major and minor constituents of *S. montana* essential oil (%) at the second cut in the second season.

Fertilization (F)	Carvacrol		Thymol		p-Cymene		γ-Terpinene		β-Caryophyllene		Linalool	
	Date of Sowing (D)											
	October	March	October	March	October	March	October	March	October	March	October	March
N0_P0	65.32	65.41	2.56	1.97	9.77	9.93	6.33	6.56	1.41	1.46	1.92	1.90
N0_P37	67.32	68.04	2.89	2.97	7.89	7.75	5.03	4.87	1.43	2.22	0.74	0.75
N0_P74	67.89	68.89	3.69	3.61	5.81	5.91	4.23	3.66	1.31	1.13	0.91	0.95
N55_P0	65.45	65.79	2.67	1.99	9.68	8.67	5.24	5.98	1.33	1.68	1.80	1.81
N55_P37	70.78	70.88	3.74	3.72	4.98	4.9	3.89	3.38	2.93	2.60	0.78	0.72
N55_P74	72.31	73.05	3.88	3.98	4.77	4.85	3.80	3.32	2.74	2.54	0.65	1.01
N110_P0	65.97	66.99	2.96	2.59	8.81	8.3	5.21	5.38	2.39	2.34	0.78	1.45
N110_P37	75.44	75.92	3.89	4.07	3.79	3.58	3.10	2.19	2.81	2.09	0.90	0.98
N110_P74	76.67	77.35	5.87	4.76	3.56	3.18	2.96	2.11	1.40	2.11	0.56	0.90

**Table 5:** Antimicrobial activity evaluated with the disc diffusion method in (mm).

Microorganism	Inhibition Zone	ATB
Gram-positive bacteria		
*Micrococcus luteus* CCM 732	8.67 ± 0.58 ^a^	29.67 ± 0.58 ^ab^
*Listeria monocytogenes* CCM 4699	8.33 ± 0.57 ^ab^	30.33 ± 0.57 ^a^
*Staphylococcus aureus* CCM 3953	9.33 ± 0.58 ^a^	28.33 ± 0.58 ^b^
Gram-negative bacteria		
*Escherichia coli* CCM 3953	6.67 ± 0.57 ^bc^	28.67 ± 0.57 ^ab^
*Salmonella enterica* subsp. *enterica* CCM 3807	6.33 ± 0.58 ^cd^	29.33 ± 0.58 ^ab^
*Yersinia enterocolitica* CCM 7204T	5.67 ± 0.59 ^cde^	29.67 ± 0.58 ^ab^
Yeasts		
*Candida albicans* CCM 8186	4.33 ± 0.58 ^e^	28.33 ± 0.58 ^b^
*Candida glabrata* CCM 8270	5.33 ± 0.58 ^cde^	29.67 ± 0.58 ^ab^
*Candida krusei* CCM 8271	4.67 ± 0.58 ^de^	29.33 ± 0.59 ^ab^
*Candida tropicalis* CCM 8223	4.33 ± 0.59 ^e^	29.67 ± 0.59 ^ab^

Data are the mean (±SD) of three samples. Different letters in each column refer to significant differences (Tukey, *p* < 0.05). ATB = Antibiotics.

**Table 6:** Minimal inhibition concentration (MIC) and minimal biofilm inhibition concentration (MBIC) of *S. montana* essential oil in μg/mL.

Microorganism	MIC_50_	MIC_90_
Gram-positive bacteria		
*Micrococcus luteus* CCM 732	3.34 ± 0.21 ^g^	3.78 ± 0.10 ^g^
*Listeria monocytogenes* CCM 4699	2.42 ± 0.23 ^h^	2.73 ± 0.14 ^h^
*Staphylococcus aureus* CCM 3953	2.19 ± 0.14 ^h^	2.35 ± 0.10 ^h^
Gram-negative bacteria		
*Escherichia coli* CCM 3953	13.16 ± 0.06 ^e^	13.37 ± 0.18 ^e^
*Salmonella enterica* subsp. *enterica* CCM 3807	17.35 ± 0.16 ^d^	17.63 ± 0.15 ^d^
*Yersinia enterocolitica* CCM 7204T	18.43 ± 0.28 ^c^	18.65 ± 0.14 ^c^
Yeasts		
*Candida albicans* CCM 8186	22.18 ± 0.25 ^b^	22.35 ± 0.07 ^b^
*Candida glabrata* CCM 8270	23.43 ± 0.10 ^a^	23.74 ± 0.22 ^a^
*Candida krusei* CCM 8271	22.24 ± 0.08 ^b^	22.43 ± 0.10 ^b^
*Candida tropicalis* CCM 8223	23.24 ± 0.18 ^a^	23.52 ± 0.13 ^a^
Biofilm forming bacteria (BFB)		
*Salmonella enterica*	4.56 ± 0.25 ^f^	4.79 ± 0.20 ^f^

Data are the mean (±SD) of three samples. Different letters in each column refer to significant differences (Tukey, *p* < 0.05).

**Table 7:** In situ analysis of the antimicrobial activity (inhibition of microbial growth %) of the vapor phase of SMEO in kohlrabi.

Food Model	Microorganisms	Concentration of EO (μg/mL)			
Kohlrabi		62.5	125	250	500
Gram-positive	*Micrococcus luteus*	85.34 ± 2.40 ^b^	65.48 ± 1.71 ^bc^	56.67 ± 1.66 ^b^	44.76 ± 1.05 ^abc^
	*Listeria monocytogenes*	83.67 ± 0.89 ^b^	63.56 ± 2.18 ^c^	55.48 ± 1.08 ^b^	42.43 ± 0.95 ^c^
	*Staphylococcus aureus*	86.46 ± 1.30 ^b^	67.43 ± 1.00 ^b^	53.25 ± 0.91 ^b^	46.65 ± 0.85 ^a^
Gram-negative	*Escherichia coli*	93.38 ± 0.83 ^a^	77.43 ± 1.50 ^a^	63.65 ± 1.67 ^a^	43.54 ± 0.99 ^bc^
	*Salmonella enterica*	94.45 ± 1.16 ^a^	75.45 ± 1.18 ^a^	65.45 ± 1.27 ^a^	44.96 ± 0.78 ^ab^
	*Yersinia enterocolitica*	96.37 ± 1.30 ^a^	74.43 ± 1.08 ^a^	66.48 ± 1.28 ^a^	46.69 ± 0.89 ^a^
Yeasts	*Candida albicans*	76.56 ± 1.62 ^c^	55.47 ± 1.00 ^d^	34.54 ± 0.49 ^cd^	15.67 ± 0.58 ^de^
	*Candida glabrata*	76.43 ± 0.98 ^c^	54.18 ± 1.08 ^d^	32.28 ± 0.82 ^d^	16.65 ± 0.45 ^de^
	*Candida krusei*	75.76 ± 0.88 ^c^	56.78 ± 0.88 ^d^	35.65 ± 1.75 ^cd^	17.54 ± 1.20 ^d^
	*Candida tropicalis*	73.43 ± 0.72 ^c^	57.77 ± 0.86 ^d^	36.56 ± 1.08 ^c^	14.76 ± 0.58 ^e^

Data are the mean (±SD) of three samples. Different letters in each column refer to significant differences (Tukey, *p* < 0.05).

**Table 8:** Insecticidal activity of SMEO against *Harmonia axyridis*.

Concentration (%)	Number of Living Individuals	Number of Dead Individuals	Insecticidal Activity (%)
100	0	100	100.00 ± 0.00
50	20	80	80.00 ± 0.00
25	30	70	70.00 ± 0.00
12.5	40	60	60.00 ± 0.00
6.25	60	40	40.00 ± 0.00
3.125	90	10	10.00 ± 0.00
Control group	100	0	0.00

## Data Availability

The original contributions presented in the study are included in the article, further inquiries can be directed to the corresponding authors.

## Supplementary Materials

The following supporting information can be downloaded at: https://www.mdpi.com/article/10.3390/biology13050328/s1, Table S1. Analysis of statistical significance (*p*-value) of sowing date and N-P fertilization for fresh weight, oil content and oil yield; Table S2. Effect of fertilization with N and P as well as their combination on the trace constituents of *S. montana* essential oil at 2nd cut in the second season during planting dates at 2nd cut in the second season; Table S3. Effect of sowing date and fertilization with N and P as well as their combination on the major chemical compound groups of *S. montana* essential oil at 2nd cut in the second season.
