# Supplementary material for: Phytochemical Characterization and Biological Activities of Essential Oil from Satureja montana L., a Medicinal Plant Grown under the Influence of Fertilization and Planting Dates
Source: Biology (Basel). 2024 May 8;13(5):328. doi: 10.3390/biology13050328 (PMC11118672; doi:10.3390/biology13050328)
Supplement: Supplementary file 1 [file biology-13-00328-s001.zip › biology-2987233-supplementary.pdf]

Table S1. Analysis of statistical significance (p-value) of sowing date and N-P fertilization for fresh weight, oil content and oil yield.

Parameter	Fertilization (F)	Date of sowing (D)	Fertilization x Date of sowing (D x F)
Fresh weight (g per plant) in the first cut	p < 0.001	p < 0.001	p < 0.001
Fresh weight (g per plant) in the second cut	p < 0.001	p < 0.001	p = 0.001
Oil content (mL/100g FW) in the first cut	p < 0.001	p < 0.001	p < 0.001
Oil content (mL/100g FW) in the second cut	p < 0.001	p < 0.001	p = 0.050
Oil yield (mL per plant FW) in the first cut	p < 0.001	p < 0.001	p < 0.001
Oil yield (mL per plant FW) in the second cut	p < 0.001	p < 0.001	p = 0.001

Table S2. Effect of fertilization with N and P as well as their combination on the trace constituents of *S. montana* essential oil at 2nd cut in the second season during planting dates at 2nd cut in the second season.

Component	N0_P0	N0_P37	N0_P74	N55_P0	N55_P37	N55_P74	N110_P0	N110_P37	N110_P74
					**October**				
α-thujene	0.21	0.24	0.22	0.24	0.26	0.21	0.41	0.20	0.19
α-pinene	0.59	0.70	0.97	0.34	0.71	0.59	0.53	0.68	0.58
β-pinene	0.58	0.68	0.57	0.80	0.49	0.86	0.77	0.44	0.90
1-octen-3-ol	0.65	0.51	0.32	0.50	0.40	0.57	0.38	0.58	0.42
α-Phellandrene	0.54	0.53	0.60	0.46	0.44	0.61	0.32	0.11	0.27
β-myrcene	0.45	0.33	0.77	0.65	0.76	0.54	0.42	0.56	0.32
α-terpinene	0.76	0.52	0.76	0.78	0.13	0.21	0.11	0.14	0.61
limonene	0.75	0.21	0.23	0.43	0.31	0.42	0.50	0.22	0.33
eucalyptol	0.17	0.25	0.21	0.22	0.42	0.20	0.12	0.58	0.46
1-terpinen-4-ol	0.60	0.45	0.38	0.71	0.62	0.97	0.88	0.87	0.85
Germacrene D	0.61	0.55	0.40	0.32	0.52	0.31	0.19	0.21	0.21
caryophyllene oxide	0.34	0.24	0.60	0.52	0.66	0.33	0.48	0.44	0.23
					**March**				
α-thujene	0.21	0.24	0.22	0.24	0.26	0.21	0.41	0.20	0.19
α-pinene	0.59	0.70	0.97	0.34	0.71	0.59	0.53	0.68	0.58
β-pinene	0.58	0.68	0.57	0.80	0.49	0.86	0.77	0.44	0.90
1-octen-3-ol	0.65	0.51	0.32	0.50	0.40	0.57	0.38	0.58	0.42
α-Phellandrene	0.54	0.53	0.60	0.46	0.44	0.61	0.32	0.11	0.27
β-myrcene	0.45	0.33	0.77	0.65	0.76	0.54	0.42	0.56	0.32
α-terpinene	0.76	0.52	0.76	0.78	0.13	0.21	0.11	0.14	0.61
limonene	0.75	0.21	0.23	0.43	0.31	0.42	0.50	0.22	0.33
eucalyptol	0.17	0.25	0.21	0.22	0.42	0.20	0.12	0.58	0.46
1-terpinen-4-ol	0.60	0.45	0.38	0.71	0.62	0.97	0.88	0.87	0.85
Germacrene D	0.61	0.55	0.40	0.32	0.52	0.31	0.19	0.21	0.21
caryophyllene oxide	0.34	0.24	0.60	0.52	0.66	0.33	0.48	0.44	0.23

Table S3. Effect of sowing date and fertilization with N and P as well as their combination on the major chemical compound groups of *S. montana* essential oil at 2nd cut in the second season.

Fertilization (F)	Monoterpene hydrocarbones		Monoterpenes oxygenated		Sesquiterpene hydrocarbones		Sesquiterpenes oxygenated	
	Date of sowing (D)							
	Oct	Mar	Oct	Mar	Oct	Mar	Oct	Mar
N0_P0	19.98	19.98	70.57	69.84	2.02	2.24	0.34	0.65
N0_P37	16.62	16.21	71.88	73.06	1.75	2.42	0.52	0.62
N0_P74	13.14	12.51	73.53	74.54	1.83	1.68	0.66	0.54
N55_P0	18.13	17.41	70.62	70.80	1.88	2.46	0.24	0.62
N55_P37	12.31	11.76	76.47	76.53	3.24	2.86	0.33	0.48
N55_P74	11.63	11.87	77.84	79.00	2.93	2.75	0.48	0.29
N110_P0	18.14	18.13	70.30	72.02	2.79	2.73	0.60	0.55
N110_P37	9.24	9.88	81.68	82.29	3.02	2.12	0.44	0.47
N110_P74	9.72	8.97	84.41	84.71	1.61	2.21	0.23	0.19
